# Treatment satisfaction and patient reported outcomes among people with opioid use disorder participating in an open‐label, non‐randomised trial of long‐acting injectable buprenorphine treatment in Australian custodial settings

**DOI:** 10.1111/dar.14005

**Published:** 2025-02-03

**Authors:** Bethany White, Sophia Little, Paul S. Haber, Jillian Roberts, Erin Nolan, Nicholas Lintzeris, Adrian J. Dunlop

**Affiliations:** ^1^ Edith Collins Centre for Translational Research in Alcohol, Drugs and Toxicology, Sydney Local Health District Sydney Australia; ^2^ Specialty of Addiction Medicine, Faculty of Medicine and Health, The University of Sydney Sydney Australia; ^3^ Drug & Alcohol Clinical Research & Improvement Network Sydney Australia; ^4^ Justice Health and Forensic Mental Health Network Sydney Australia; ^5^ Drug & Alcohol Clinical Services, Hunter New England Local Health District Newcastle Australia; ^6^ Hunter Medical Research Institute Newcastle Australia; ^7^ Drug and Alcohol Services, South Eastern Sydney Local Health District Sydney Australia; ^8^ Faculty of Health and Medicine, University of Newcastle Newcastle Australia; ^9^ Healthcare Transformation Research Program, Hunter Medical Research Institute Newcastle Australia

**Keywords:** long‐acting injectable buprenorphine, medication satisfaction, opioid use disorder, prison, patient reported outcomes

## Abstract

**Introduction:**

A trial of long‐acting injectable buprenorphine (LAIB) in Australian prisons allowed examination of treatment satisfaction and patient‐reported outcomes.

**Methods:**

UNLOC‐T was a 16‐week non‐randomised open‐label study. Men and women aged ≥18 years with moderate/severe DSM‐5 opioid use disorder currently serving a custodial sentence ≥6 months were recruited. Participants not in opioid agonist treatment (OAT) commenced LAIB (*n* = 67); those already stable on oral methadone treatment were recruited to a comparison arm (*n* = 62). The Treatment Satisfaction Questionnaire for Medication (TSQM), Patient Satisfaction Visual Analogue Scale (PS‐VAS) and Treatment Burden Questionnaire assessed treatment satisfaction; the Kessler Psychological Distress Scale (K10), 12‐Item Short Form Health Survey (SF‐12) and the Australian Treatment Outcomes Profile (ATOP) measured mental health, physical health and quality of life.

**Results:**

Among participants receiving LAIB, TSQM global satisfaction scores significantly increased from 68.2 (SD 16.6) to 77.0 (SD 18.4) by week 16 (*p* = 0.0041), as did satisfaction measured by the PS‐VAS (62.5 [SD 29.2] vs. 79.4 [SD 25.5], *p* = 0.0005). Statistically significant improvements between baseline and week 16 were also observed for K10, SF‐12 (total) and SF‐12 (mental health) scores. By the end of the study, ‘successful’ treatment outcomes were observed in the ATOP domains of psychological health (84%), physical health (80%) and quality of life (86%).

**Discussion and Conclusions:**

Participants inducted and stabilised on LAIB reported high treatment satisfaction and improved health and wellbeing. Results suggest LAIB is acceptable to people with opioid use disorder in custody, supporting scaleup of this medication to increase coverage of OAT in these settings.

Trial registration: https://www.anzctr.org.au ACTRN12618000942257.

## 1 INTRODUCTION

Opioid use disorder (OUD) is an ongoing public health challenge, contributing to global health burden and associated with high rates of overdose deaths [1]. Opioid agonist treatment (OAT) including methadone and buprenorphine are effective and widely utilised [2], including evidence of effectiveness for more recently approved long‐acting injectable buprenorphine (LAIB) formulations [3, 4, 5]. OUD is common in custodial populations [6], and is associated with elevated risk of mortality from overdose while in custody and following release [7]. Injecting drug use and needle and syringe‐sharing during incarceration has been documented globally [8] and are key drivers of HIV, hepatitis C virus and other infections in custody in the context of no access to sterile needle and syringes [9]. Evidence of the effectiveness of daily oral OAT in custody is well established [10], with evidence emerging for long‐acting injectable forms in this setting [11].

High levels of patient satisfaction with LAIB [4, 12, 13, 14] and improvements in patient reported outcomes including mental health, addiction severity and craving [15, 16] have been described in community settings. A qualitative study of people recently released from New York City jails switched from sublingual to LAIB on release indicated acceptable levels satisfaction [17] however, to our knowledge, patient reported outcomes of people with OUD being treated with LAIB while in custody have yet to be documented.

A trial of LAIB for the treatment of opioid dependence in Australian correctional centres—the UNLOC‐T study—was designed to address the primary objectives of feasibility and safety [18], but included evaluation of patient‐reported outcomes as secondary objectives. The current paper presents the secondary outcomes including: (i) examination of satisfaction with LAIB over the trial period, including perceived treatment burden; and (ii) changes in patient‐reported measures of quality of life, psychological health and physical health in patients initiated to LAIB treatment.

## 2 METHODS

### 2.1 Ethical conduct and approval

The study was approved by Justice Health and Forensic Mental Health (JHFMHN) and the Aboriginal Health and Medical Research Council Human Research Ethics Committees (Protocol JH File No G561/17 & HREC/18/JH/3), as well as the Corrective Services NSW Ethics Committee. Participation was voluntary and written, informed consent was obtained from all participants prior to the conduct of any study procedure.

### 2.2 Study design and setting

The UNLOC‐T study was a 16‐week non‐randomised open‐label trial conducted in 2018–2019 [18]. Study participants were adults in New South Wales (NSW) public prisons seeking treatment for moderate to severe OUD, who had a minimum 6 months left on their sentence to allow for at least 4 months of treatment in custody and were medically fit to receive OAT, but who were not otherwise eligible to receive OAT via JHFMHN treatment protocols available at the time for ‘Priority’ (e.g., HIV positive people, pregnant women) or ‘Fast Track’ (e.g., people with significant current medical illness) access.

Adult men and women were recruited and followed‐up in seven correctional centres across six metropolitan and rural areas of NSW with a range of security classifications. Participants were initiated onto buprenorphine treatment with a 4 mg test‐dose of sublingual buprenorphine‐naloxone film (day 0) and observed for 1 h to confirm opioid tolerability. Treatment with LAIB was initiated with a single dose of a weekly 16 mg injection (day 1), with patients subsequently administered three weekly LAIB injections followed by three monthly injections over 16 weeks with doses individually titrated. The comparator group—patients who entered custody on oral methadone which was continued and therefore stable on treatment—reflected ‘treatment as usual’, with outcomes reported to provide context [18].

### 2.3 Patient‐reported outcome measures

Surveys documenting treatment satisfaction, treatment burden and health‐related outcomes were administered face‐to‐face by trained researchers in custodial health clinics at baseline (day 1, immediately following first weekly injection), week 4 (immediately following first monthly injection) and week 16 (4 weeks after the third monthly injection).

Treatment Satisfaction Questionnaire for Medication (TSQM, v14.1) comprises 14 items across four categories: convenience, effectiveness, side effects and global satisfaction relating to current experience of medication [19]. Except for item 4 (presence of side effects; yes or no) all items are scored on 5‐ or 7‐point Likert scales. The scores for each domain are computed by adding the TSQM items in each domain and then transforming the composite score into a value ranging from 0 to 100, where higher scores indicate greater satisfaction, and a score ≥80 considered high‐level satisfaction [20]. Patient satisfaction was also measured on a visual analogue scale (PS‐VAS), comprising a 10 cm line with two end points 0 (‘not at all’) and 100 (extremely) and the instruction ‘select a point on the line to represent how satisfied you are with your treatment’. The Treatment Burden Questionnaire (TBQ) comprises 13 items scored from 0 (not a problem) to 10 (a big problem), summed to provide a global score (range 1–130) with higher scores indicating greater burden [21].

The Kessler Psychological Distress Scale (K10) is a 10‐item questionnaire designed to measure psychological distress [22]. Scores out of 50 are categorised as indicating low (10–15), moderate (16–21), high (22–29) or very high (30–50) distress. The 12‐item short form survey (SF‐12) is reported in two summary scores—physical health and mental health, as well as a total score [23]. The lower the score the worse a patient is functioning in each area. Scores are weighted to a normal distribution (mean = 50). A score <40 is considered impairment in that domain.

Physical health, psychological health and quality‐of‐life were also self‐rated on a scale from 1 (poor) to 10 (good) using items from the Australian Treatment Outcomes Profile (ATOP), with scores ≤5 categorised as impairment in each of these domains (‘cases’), and scores ≥6 as ‘normal’/well (‘non‐cases’) [24]. Each treatment episode was assessed for ‘successful treatment outcome’ using the ‘Clinical Outcomes and Quality Indicators metric’ [25]. Treatment was considered ‘successful’ if cases increased their score by ≥2, or if non‐cases improved or maintained their score (i.e., did not deteriorate) [25].

### 2.4 Statistical analysis

Intention‐to‐treat analyses were performed using SAS 9.4, with observations for those who ceased treatment and for those with missing observations at week 4 and 16 included in the models. Outcomes were analysed using general linear mixed effect regression models for time (baseline, week 16) and a random intercept for subject to account for repeated measures and robust standard errors (HC3) to account for any non‐constant variance [26]. Changes in scores within the LAIB group over the study period are presented with 95% confidence intervals (CIs) and *p*‐values; scores for the methadone group are presented for comparative purposes only.

TSQM subdomains (convenience, effectiveness, global satisfaction but not side effects, which was modelled [yes/no]), PS‐VAS, TBQ, ATOP subdomains (physical, psychological, quality‐of‐life) and SF‐12 (total, physical health and mental health) scores were modelled using the normal distribution. K10 scores were categorised into the four clinically relevant groups—‘1’ well, ‘2’ likely mild mental illness, ‘3’ likely moderate mental illness and ‘4’ likely severe mental illness—with the groups modelled using ‘K10 group’ as the outcome. The multinomial distribution with a cumulative logit link was used; the model was therefore modelling the probability of having a lower ordered K10 group.

## 3 RESULTS

Of the 67 patients initiated onto LAIB, 64 (96%) completed week 4 and 59 (88%) completed week 16 surveys; 5 LAIB treatment dropouts were voluntary, 6 were involuntary (transfer to a non‐trial site or early release from custody) and 1 participant retained in treatment refused the 16‐week survey. Retention in treatment at 16 weeks was 56 of 67 (84%); 4 participants who completed surveys after ceasing treatment were included in the current intention‐to‐treat analysis.

As described previously [18] similar proportions of patients in the LAIB and methadone groups were male (82% vs. 85%, *p* = 0.602), Australian‐born (94% vs. 94%, *p* = 0.910) and identified as Aboriginal and/or Torres Strait Islander people (42% vs. 34%, *p* = 0.354), although patients recruited to the LAIB arm were significantly younger (34 [SD = 7.5] vs. 38 [SD = 8.9] years, *p* = 0.004) and were less likely to report not having completed year 10/secondary education (46% vs. 68%, *p* = 0.014). At week 16, mean LAIB dose was 136 mg (SD 23 mg; range 64–160 mg) and average methadone dose was 94 mg (SD 40 mg).

By week 16, the TSQM global satisfaction mean score had increased from 68.2 (SD 16.6) to 77.0 (SD 18.4) (mean difference 9.0; 95% CI 2.9, 15.0, *p* = 0.0041). Improved scores between baseline and week 16 were also observed in the secondary TSQM domains of effectiveness and side effects (Table 1). Satisfaction with LAIB as measured by the PS‐VAS also increased, with participants reporting a mean difference of 18 (95% CI 8.0, 27.3, *p* = 0.0005) increase from baseline, while global TBQ scores remained stable. Although not statistically assessed, mean scores in the methadone group for the three satisfaction measures appeared to remain stable over the study period.

**TABLE 1:** Mean score changes in Treatment Satisfaction Questionnaire for Medication (TSQM), Patient Satisfaction Visual Analog Scale (PS‐VAS), Treatment Burden Questionnaire (TBQ), Kessler Psychological Distress Scale (K‐10) and Short Form Health Survey (SF‐12) among participants receiving long‐acting injectable buprenorphine (LAIB).

	Score, mean (SD)		Mean score change in LAIB participants			
	Methadone^a^	LAIB	Mean difference between baseline and week 16 scores			
			Estimate	Lower	Upper	p‐value
TSQM global satisfaction						
Baseline	*n* = 62 *69.2 (19.7)*	*n* = 53 68.2 (16.6)				
Week 4	*n* = 62 *73.2 (19.1)*	*n* = 64 75.8 (19.7)				
Week 16	*n* = 60 *72.9 (20.7)*	*n* = 59 77.0 (18.4)	9.0	2.9	15.0	0.0041
TSQM effectiveness						
Baseline	*n* = 62 *71.7 (11.3)*	*n* = 26 67.1 (9.7)				
Week 4	*n* = 62 *73.8 (12.8)*	*n* = 64 70.8 (17.8)				
Week 16	*n* = 60 *74.3 (15.1)*	*n* = 59 74.3 (14.4)	7.9	2.3	13.6	0.0067
TSQM convenience						
Baseline	*n* = 62 *71.4 (12.9)*	*n* = 58 73.7 (11.7)				
Week 4	*n* = 62 *74.4 (12.8)*	*n* = 64 73.9 (13.5)				
Week 16	*n* = 60 *70.5 (12.8)*	*n* = 59 75.8 (14.8)	2.1	−2.2	6.3	0.3378
TSQM side effects						
Baseline	*n* = 62 *89.1 (22.6)*	*n* = 58 97.4 (10.4)				
Week 4	*n* = 62 *94.1 (16.1)*	*n* = 64 92.0 (15.9)				
Week 16	*n* = 60 *90.0 (19.4)*	*n* = 59 87.7 (24.8)	1.3^b^	1.1	1.5	0.0035
PS‐VAS						
Baseline	*n* = 62 *76.1 (22.0)*	*n* = 62 62.5 (29.2)				
Week 4	*n* = 62 *77.9 (20.9)*	*n* = 64 76.2 (24.6)				
Week 16	*n* = 60 *79.9 (20.3)*	*n* = 59 79.4 (25.5)	17.6	8.0	27.3	0.0005
TBQ global score						
Baseline	*n* = 62 *16.1 (18.4)*	*n* = 67 5.6 (9.0)				
Week 4	*n* = 62 *15.4 (17.1)*	*n* = 64 6.3 (8.7)				
Week 16	*n* = 60 *20.1 (19.6)*	*n* = 59 8.3 (10.8)	2.5	−1.2	6.2	0.1831
K10 score						
Baseline	*n* = 62 *17.8 (7.7)*	*n* = 67 17.9 (7.1)				
Week 4	*n* = 62 *16.9 (7.6)*	*n* = 64 14.4 (4.8)				
Week 16	*n* = 60 *16.9 (7.0)*	*n* = 59 13.8 (5.3)	8.2^c^	2.4	28.5	0.0012
SF‐12 total						
Baseline	*n* = 62 *77.9 (13.8)*	*n* = 67 77.7 (14.2)				
Week 4	*n* = 62 *79.1 (13.3)*	*n* = 64 84.0 (11.7)				
Week 16	*n* = 60 *78.8 (13.7)*	*n* = 59 83.9 (13.5)	5.9	1.8	10.2	0.0059
SF‐12 mental health						
Baseline	*n* = 62 *45.7 (12.7)*	*n* = 67 45.7 (10.3)				
Week 4	*n* = 62 *47.6 (10.7)*	*n* = 64 49.9 (9.8)				
Week 16	*n* = 60 *47.3 (10.8)*	*n* = 59 50.8 (10.3)	4.9	1.4	8.4	0.0059
SF‐12 physical health						
Baseline	*n* = 62 *47.6 (8.7)*	*n* = 67 48.0 (7.4)				
Week 4	*n* = 62 *46.7 (7.8)*	*n* = 64 48.4 (5.4)				
Week 16	*n* = 60 *46.9 (7.9)*	*n* = 59 47.4 (5.8)	−0.6	−2.8	1.6	0.6081

*Note*: Italics denote results for the metadone arm are provided for comparision only,

^a^ Methadone scores presented for comparative purposes only.

^b^ Odds ratio: Due to the small number of respondents reporting any side effects, the side effects satisfaction scale was dichotomised to complete satisfaction (100 points out of 100) or not complete satisfaction (<100/100).

^c^ Cumulative odds ratio.

Psychological distress (K10 scores) significantly decreased from baseline to week 16 (OR 8.2 [95% CI 2.4, 28.5], *p* = 0.0012) (Table 1). Further, Total SF‐12 scores significantly improved over time (mean difference 5.9 [95% CI 1.8, 10.2], *p* = 0.0059), with improvements observed on the SF‐12 subscale mental but not physical health (Table 1). In contrast, although not statistically assessed, again mean scores on these measures of mental and physical heath appeared to remain stable in the methadone group.

Differences in ATOP psychological scores by week 16 compared to baseline suggested that 84% of patients receiving LAIB had a successful treatment outcome (i.e., had maintained ‘normal’ psychological health or improved it) (Table 2). Changes in the physical health scores of 80% of the LAIB group suggested a successful treatment outcome by week 16, while changes in quality‐of‐life scores indicated that by week 16, 86% were successful. Smaller proportions failed to achieve a favourable outcome (either continued in poor health and/or deteriorated) across domains (Table 2).

**TABLE 2:** Changes in psychological health, physical health and quality‐of‐life as measured by the Australian Treatment Outcomes Profile by week 16 among participants receiving long‐acting injectable buprenorphine (LAIB).

	Score, mean (SD)	Mean score changes in LAIB participants				Clinical outcomes and quality indicators – Treatment outcome				
	LAIB	Mean difference between baseline and week 16 scores				Successful treatment outcome (‘maintain‐non‐impaired’ + ‘improved’ scores)			Deteriorated (score dropped ≥2 pts)	Maintained‐impaired (baseline score ≤ 5pts, improved <1 pts)
		Estimate	Lower	Upper	*p*‐value	Maintained‐non‐impaired^a^	Improved^b^	Total		
Psychological health										
Baseline (*n* = 67)	7.5 (1.9)									
Week 4 (*n* = 64)	7.8 (1.9)									
Week 16 (*n* = 59)	7.9 (1.7)	0.3	−0.2	0.7	0.2384	35 (63%)	12 (21%)	47 (84%)	8 (14%)	1 (2%)
Physical health										
Baseline (*n* = 67)	7.5 (2.0)									
Week 4 (*n* = 64)	7.7 (1.9)									
Week 16 (*n* = 59)	7.9 (1.5)	0.5	0.03	0.9	0.0354	33 (59%)	12 (21%)	45 (80%)	5 (9%)	6 (11%)
Quality of life										
Baseline (*n* = 67)	7.2 (1.8)									
Week 4 (*n* = 64)	8.0 (1.7)									
Week 16 (*n* = 59)	8.1 (1.6)	0.8	0.4	1.2	0.0001	33 (59%)	15 (27%)	48 (86%)	3 (5%)	5 (9%)

^a^ Baseline score ≥6 pts; scored dropped <1 pts.

^b^ Score improved ≥2 pts.

## 4 DISCUSSION

This study found high levels of treatment satisfaction and improved patient‐reported outcomes among people with OUD inducted and stabilised onto LAIB during a 16‐week trial in custodial settings, despite 97% experiencing at least one treatment emergent adverse event as previously reported [16]. Significant improvements were observed in global medication satisfaction scores using the TSQM (including global satisfaction, effectiveness and side effects subdomain scores), and on the single‐item patient satisfaction VAS score. Significant improvements were also reported by participants in psychological health (K10, SF‐12 and ATOP), quality of life (Total SF‐12 and ATOP), and physical health (ATOP). In contrast, mean scores across all instruments appeared to remain stable in the methadone arm.

High levels of satisfaction with LAIB treatment have been previously reported. An Australian community‐based RCT found that compared to patients on sublingual buprenorphine, patients on LAIB reported high treatment satisfaction at 24 weeks [12]. A US trial found improved satisfaction over time with another LAIB formulation at 12 months [15]. Qualitative interviews with participants in Australian trials [16, 27] also identified favourable experiences of LAIB treatment. Our study is the first we are aware of to report satisfaction with LAIB for OUD treatment while in the prison environment, adding to the growing literature on patient perceptions of long‐acting injectable OAT formulations and underscoring the importance of assessing patient‐reported outcomes when comparing treatment options for OUD as in other areas of health care [28].

By week 16, TSQM global satisfaction mean score significantly increased, as did PS‐VAS scores, possibly a reflection of treatment stabilisation. At week 16 participants had mean global TSQM score of 77.0, close to the cut‐off of 80 previously defined as highly satisfied [20]. TSQM convenience domain scores remained high; suggesting convenience of a monthly treatment may be greater than a daily OAT even in the prison context, where daily dosing may impact patients' ability to engage in other activities. This finding of high convenience is consistent with the community‐based RCT comparing weekly/monthly injectable buprenorphine dosing to daily sublingual buprenorphine [12].

The perceived burden of a treatment can impact retention and perception of the treatment's value [21]. TBQ scores of participants receiving weekly then monthly LAIB treatment remained stable over the study period; however, a very low score of 8.34 (out of possible 130, where lower scores indicate lower burden) by week 16 was observed. Convenience, cost, social factors, stigma as well as medication burden are commonly identified barriers to ongoing treatment engagement and may reduce retention in otherwise successful treatment regimens [29]. Given LAIB is administered more discreetly (e.g., in a clinic room rather than a dosing queue) and less frequently than other forms of OAT, it may reduce stigma from corrections staff as reported previously [30] and save time, potentially improving retention for people treated in prison. Cost is also an important consideration for both providers and patients. In this setting there was no cost to patients for either form of OAT whereas provider costs include health care and custodial staff. We have previously reported that LAIB was the least costly form of OAT to deliver in this setting [31].

People in custody have significantly poorer mental and physical health than the general population [32]. Nevertheless, improvements were observed across multiple mental health and quality‐of‐life measures (K10, SF‐12 and ATOP) in patients receiving LAIB. Overall, improvements reported by patients in the LAIB arm by week 16 compared to baseline suggest benefit to those initiated onto it. In this regard, it is relevant to note increasing use of this form of OAT in custodial settings worldwide [11].

Limitations of the study included no matched control group, but ethical concerns precluded randomisation of patients given the absence of therapeutic equipoise. Accordingly, we selected patients who were stable on methadone as a comparison group. The study was not powered to detect between‐group differences for secondary outcomes but was adequate to identify the main effects [18]. Comparison of patient reported outcomes with community populations is also limited by many differences between custodial and community environments and limitations with available instruments including lack of validation within the prison context, clinical relevance based on sample size and study duration. Further, given the lack of alternative treatment options, there was potential for artificial inflation of reported levels of satisfaction among the LAIB group. Finally, as baseline satisfaction was measured soon after initial LAIB administration, some participants were unable to answer the TSQM items in the effectiveness domain.

Limitations notwithstanding, results suggest that LAIB was an acceptable form of OAT in the correctional setting, with health improvements observed and possibly lower mental distress once patients are stabilised. This form of OAT was also preferred by health care and custodial providers [33] and was found to be less costly due to reductions in staff resourcing [31]. The current findings are particularly important given that provider preference could be sufficient to expand treatment in this setting, without due consideration of patient perspectives. We conclude that LAIB was an acceptable form of OAT to patients in custody. In light of favourable costings, there is potential to scale up and increase coverage of OAT in these settings while maintaining patient safety and satisfaction with treatment. However, we also note the rapid uptake of LAIB in custodial settings from 2000 [34] alongside the growth of the total number of people in opioid treatment in Australia [35]. If the custodial treatment system is initiating more people on OAT, this will have an impact on the number referred for ongoing treatment in the community, which needs to be adequately resourced.

## AUTHOR CONTRIBUTIONS

Each author certifies that their contribution to this work meets the standards of the International Committee of Medical Journal Editors.

## CONFLICT OF INTEREST STATEMENT

Adrian J. Dunlop reports grants from Camurus AB and Indivior to conduct clinical studies with buprenorphine formulations to HNELHD, which employs Adrian J. Dunlop. Paul S. Haber reports grants from Camurus AB and Indivior to conduct clinical studies with buprenorphine products and has served on advisory boards for Lundbeck, Indivior, AbbVie and Gilead. Nicholas Lintzeris reports grants from Camurus AB to conduct sponsored and investigator‐led clinical studies with Buvidal and has received honoraria from Camurus AB for presenting at international conferences. All other authors declare no competing interests.

## Data Availability

The data that support the findings of this study are available from the corresponding author upon reasonable request.

## References

[1] Degenhardt L , Grebely J , Stone J , Hickman M , Vickerman P , Marshall BDL , et al. Global patterns of opioid use and dependence: harms to populations, interventions, and future action. Lancet. 2019;394:1560–1579. 31657732 10.1016/S0140-6736(19)32229-9 PMC7068135

[2] Volkow ND , Blanco C . Medications for opioid use disorders: clinical and pharmacological considerations. J Clin Invest. 2020;130:10–13. 31763992 10.1172/JCI134708 PMC6934219

[3] Marsden J , Kelleher M , Gilvarry E , Mitcheson L , Bisla J , Cape A , et al. Superiority and cost‐effectiveness of monthly extended‐release buprenorphine versus daily standard of care medication: a pragmatic, parallel‐group, open‐label, multicentre, randomised, controlled, phase 3 trial. EClinical Medicine. 2023;66:102311. 10.1016/j.eclinm.2023.102311 PMC10692661 38045803

[4] Frost M , Bailey GL , Lintzeris N , Strang J , Dunlop A , Nunes EV , et al. Long‐term safety of a weekly and monthly subcutaneous buprenorphine depot (CAM2038) in the treatment of adult out‐patients with opioid use disorder. Addiction. 2019;114:1416–1426. 31013390 10.1111/add.14636 PMC6771955

[5] Lofwall MR , Walsh SL , Nunes EV , Bailey GL , Sigmon SC , Kampman KM , et al. Weekly and monthly subcutaneous buprenorphine depot formulations vs daily sublingual buprenorphine with naloxone for treatment of opioid use disorder: a randomized clinical trial. JAMA Intern Med. 2018;178:764–773. 29799968 10.1001/jamainternmed.2018.1052 PMC6145749

[6] Fazel S , Yoon IA , Hayes AJ . Substance use disorders in prisoners: an updated systematic review and meta‐regression analysis in recently incarcerated men and women. Addiction. 2017;112:1725–1739. 28543749 10.1111/add.13877 PMC5589068

[7] Merrall ELC , Kariminia A , Binswanger IA , Hobbs MS , Farrell M , Marsden J , et al. Meta‐analysis of drug‐related deaths soon after release from prison. Addiction. 2010;105:1545–1554. 20579009 10.1111/j.1360-0443.2010.02990.x PMC2955973

[8] Moazen B , Saeedi Moghaddam S , Silbernagl MA , Lotfizadeh M , Bosworth RJ , Alammehrjerdi Z , et al. Prevalence of drug injection, sexual activity, tattooing, and piercing among prison inmates. Epidemiol Rev. 2018;40:58–69. 29860343 10.1093/epirev/mxy002

[9] Dolan K , Wirtz AL , Moazen B , Ndeffo‐Mbah M , Galvani A , Kinner SA , et al. Global burden of HIV, viral hepatitis, and tuberculosis in prisoners and detainees. Lancet. 2016;388:1089–1102. 27427453 10.1016/S0140-6736(16)30466-4

[10] Moore KE , Roberts W , Reid HH , Smith KMZ , Oberleitner LMS , McKee SA . Effectiveness of medication‐assisted treatment for opioid use in prison and jail settings: a meta‐analysis and systematic review. J Subst Abus Treat. 2019;99:32–43. 10.1016/j.jsat.2018.12.003 PMC6391743 30797392

[11] Russell C , George TP , Chopra N , Le Foll B , Matheson FI , Rehm J , et al. Feasibility and effectiveness of extended‐release buprenorphine (XR‐BUP) among correctional populations: a systematic review. Am J Drug Alcohol Abuse. 2024;50:567–586. 38940929 10.1080/00952990.2024.2360984

[12] Lintzeris N , Dunlop AJ , Haber PS , Lubman DI , Graham R , Hutchinson S , et al. Patient‐reported outcomes of treatment of opioid dependence with weekly and monthly subcutaneous depot vs daily sublingual buprenorphine: a randomized clinical trial. JAMA Netw Open. 2021;4:e219041. 33970256 10.1001/jamanetworkopen.2021.9041 PMC8111483

[13] Allen E , Samadian S , Altobelli G , Johnson J , Holmwood C . Exploring patient experience and satisfaction with depot buprenorphine formulations: a mixed‐methods study. Drug Alcohol Rev. 2023;42:791–802. 36788357 10.1111/dar.13616

[14] Parsons G , Ragbir C , D'Agnone O , Gibbs A , Littlewood R , Hard B . Patient‐reported outcomes, experiences and satisfaction with weekly and monthly injectable prolonged‐release buprenorphine. Subst Abus Rehabil. 2020;11:41–47. 10.2147/SAR.S266838 PMC7648142 33173372

[15] Ling W , Nadipelli VR , Solem CT , Ronquest NA , Yeh Y‐C , Learned SM , et al. Effects of monthly buprenorphine extended‐release injections on patient‐centered outcomes: a long‐term study. J Subst Abus Treat. 2020;110:1–8. 10.1016/j.jsat.2019.11.004 31952623

[16] Barnett A , Savic M , Lintzeris N , Bathish R , Arunogiri S , Dunlop AJ , et al. Tracing the affordances of long‐acting injectable depot buprenorphine: a qualitative study of patients' experiences in Australia. Drug Alcohol Depend. 2021;227:108959. 34450472 10.1016/j.drugalcdep.2021.108959

[17] Cheng A , Badolato R , Segoshi A , McDonald R , Malone M , Vasudevan K , et al. Perceptions and experiences toward extended‐release buprenorphine among persons leaving jail with opioid use disorders before and during COVID‐19: an in‐depth qualitative study. Addict Sci Clin Pract. 2022;17:4. 35093164 10.1186/s13722-022-00288-4 PMC8800291

[18] Dunlop AJ , White B , Roberts J , Cretikos M , Attalla D , Ling R , et al. Treatment of opioid dependence with depot buprenorphine (CAM2038) in custodial settings. Addiction. 2022;117(2):382–391. 34184798 10.1111/add.15627 PMC9291502

[19] Atkinson MJ , Sinha A , Hass SL , Colman SS , Kumar RN , Brod M , et al. Validation of a general measure of treatment satisfaction, the treatment satisfaction questionnaire for medication (TSQM), using a national panel study of chronic disease. Health Qual Life Outcomes. 2004;2:12. 14987333 10.1186/1477-7525-2-12 PMC398419

[20] Radawski C , Genovese MC , Hauber B , Nowell WB , Hollis K , Gaich CL , et al. Patient perceptions of unmet medical need in rheumatoid arthritis: a cross‐sectional survey in the USA. Rheumatol Ther. 2019;6:461–471. 31385264 10.1007/s40744-019-00168-5 PMC6702617

[21] Tran VT , Harrington M , Montori VM , Barnes C , Wicks P , Ravaud P . Adaptation and validation of the treatment burden questionnaire (TBQ) in English using an internet platform. BMC Med. 2014;12:109. 24989988 10.1186/1741-7015-12-109 PMC4098922

[22] Kessler RC , Andrews G , Colpe LJ , Hiripi E , Mroczek DK , Normand SL , et al. Short screening scales to monitor population prevalences and trends in non‐specific psychological distress. Psychol Med. 2002;32:959–976. 12214795 10.1017/s0033291702006074

[23] Ware J Jr , Kosinski M , Keller SD . A 12‐item short‐form health survey: construction of scales and preliminary tests of reliability and validity. Med Care. 1996;34:220–233. 8628042 10.1097/00005650-199603000-00003

[24] Mammen K , Mills L , Deacon RM , Bruno R , Dunlop A , Holmes J , et al. Determining clinical cutoff scores for the Australian treatment outcomes profile psychological health, physical health and quality of life questions. Drug Alcohol Rev. 2022;41:106–113. 34189792 10.1111/dar.13346

[25] Lintzeris N , Deacon R , Mills L , Bruno R , Black E , Holmes J , et al. The Australian Treatment Outcomes Profile (ATOP) manual 2: research and service evaluation. Available from: www.seslhd.health.nsw.gov.au/australian‐treatment‐outcomes‐profile. Retrieved 25 July 2023

[26] MacKinnon JG , White H . Some heteroskedasticity consistent covariance matrix estimators with improved finite sample properties. Kingston, Ontario: Queen's University, Department of Economics; 1983.

[27] Treloar C , Lancaster K , Gendera S , Rhodes T , Shahbazi J , Byrne M , et al. Can a new formulation of opiate agonist treatment alter stigma?: place, time and things in the experience of extended‐release buprenorphine depot. Int J Drug Policy. 2022;107:103788. 35816790 10.1016/j.drugpo.2022.103788

[28] Compton WM , Volkow ND . Extended‐release buprenorphine and its evaluation with patient‐reported outcomes. JAMA Netw Open. 2021;4:e219708. 33970262 10.1001/jamanetworkopen.2021.9708

[29] Tran V‐T , Montori V , Ravaud P . Is my patient overwhelmed? determining thresholds for acceptable burden of treatment using data from the ComPaRe e‐cohort. Mayo Clin Proc. 2020;95:504–512. 31619365 10.1016/j.mayocp.2019.09.004

[30] Russell C , Nafeh F , Pang M , MacDonald SF , Derkzen D , Rehm J , et al. Opioid agonist treatment (OAT) experiences and release plans among federally incarcerated individuals with opioid use disorder (OUD) in Ontario, Canada: a mixed‐methods study. BMC Public Health. 2022;22:436. 35246083 10.1186/s12889-022-12685-0 PMC8897889

[31] Ling R , White B , Roberts J , Cretikos M , Howard MV , Haber PS , et al. Depot buprenorphine as an opioid agonist therapy in New South Wales correctional centres: a costing model. BMC Health Serv Res. 2022;22:1326. 36348369 10.1186/s12913-022-08687-8 PMC9644557

[32] Australian Institute of Health and Welfare . The health of Australia's prisoners 2018. Canberra: Australian Institute of Health and Welfare; 2019.

[33] Little SC , White B , Moensted M , Butler K , Howard M , Roberts J , et al. Health and correctional staff acceptability of depot buprenorphine in NSW prisons. Int J Drug Policy. 2023;114:103978. 36870227 10.1016/j.drugpo.2023.103978

[34] Bharat C , Chidwick K , Gisev N , Farrell M , Ali R , Degenhardt L . Trends in use of medicines for opioid agonist treatment in Australia, 2013‐2022. Int J Drug Policy. 2024;123:104255. 38029481 10.1016/j.drugpo.2023.104255

[35] Australian Institute of Health and Welfare . National opioid pharmacotherapy statistics annual data collection. Canberra: Australian Institute of Health and Welfare; 2024. Available from: https://www.aihw.gov.au/reports/alcohol‐other‐drug‐treatment‐services/national‐opioid‐pharmacotherapy‐statistics/contents/summary

